# Is my visualization better than yours? Analyzing factors modulating exponential growth bias in graphs

**DOI:** 10.3389/fpsyg.2023.1125810

**Published:** 2023-02-16

**Authors:** Gerda Ana Melnik-Leroy, Linas Aidokas, Gintautas Dzemyda, Giedrė Dzemydaitė, Virginijus Marcinkevičius, Vytautas Tiešis, Ana Usovaitė

**Affiliations:** Institute of Data Science and Digital Technologies, Vilnius University, Vilnius, Lithuania

**Keywords:** cognitive bias, exponential growth, graph perception, logarithmic scaling, mathematical literacy, dual-process model

## Abstract

Humans tend to systematically underestimate exponential growth and perceive it in linear terms, which can have severe consequences in a variety of fields. Recent studies attempted to examine the origins of this bias and to mitigate it by using the logarithmic vs. the linear scale in graphical representations. However, they yielded conflicting results as to which scale induces more perceptual errors. In the current study, in an experiment with a short educational intervention, we further examine the factors modulating the exponential bias in graphs and suggest a theoretical explanation for our findings. Specifically, we test the hypothesis that each of the scales can induce misperceptions in a particular context. In addition to this, we explore the effect of mathematical education by testing two groups of participants (with a background in humanities vs. formal sciences). The results of this study confirm that when used in an inadequate context, these scales can have a dramatic effect on the interpretation of visualizations representing exponential growth. In particular, while the log scale leads to more errors in graph description tasks, the linear scale misleads people when they have to make predictions on the future trajectory of exponential growth. The second part of the study revealed that the difficulties with both scales can be reduced by means of a short educational intervention. Importantly, while no difference between participants groups was observed prior to the intervention, participants with a better mathematical education showed a stronger learning effect at posttest. The findings of this study are discussed in light of a dual-process model.

## 1. Introduction

Exponential growth is intrinsic to a large number of phenomena, ranging from the proliferation of microorganisms in biology, to compounding interests in economics or the nuclear chain reaction in physics (Lamarsh, 1983; Marr, 1991; Levy and Tasoff, 2017). Nevertheless, a growing body of literature confirms the difficulty of correctly perceiving this type of growth (Wagenaar and Sagaria, 1975; Wagenaar and Timmers, 1979). Specifically, people tend to systematically underestimate it and perceive it in terms of linear growth (Levy and Tasoff, 2017). This perceptual error has been termed ‘the exponential growth bias’. Importantly, a biased perception of exponential growth has been shown to impact real-world behavior (Christandl and Fetchenhauer, 2009; Levy and Tasoff, 2016) and it recently attracted much attention due to its relevance in the context of the Covid-19-pandemic. Namely, the infection rate of this virus follows an exponential trend, as according to estimations, the number of positive Covid-19 cases doubles every 3 days (Pellis et al., 2021). This growth has often been shown graphically in mainstream media (Engledowl and Weiland, 2021). Unfortunately, both the general public and government officials tended to misperceive it and to underestimate the risks and the severity of the disease (Gaissmaier, 2019; Lammers et al., 2020; Podkul et al., 2020). In particular, when asked to intuitively predict the number of COVID-19 cases in the future, many people underestimated how fast this value will increase (Banerjee and Majumdar, 2020; Jäckle and Ettensperger, 2021). They tended to think that the infections increase by a constant amount over each time interval (as is the case in linear growth), whereas in reality, exponential growth accelerates over time. Thus, as the quantity increases, so does that rate at which it grows: the more infections occur at the beginning of a disease outbreak, the more people will get infected. Nevertheless, people not only fail to perceive this growth, but they are also unaware of their errors (Cordes et al., 2019) and are even overconfident in their ability to deal with exponential growth (Levy and Tasoff, 2017). Growing evidence points to the fact that this has directly impacted the compliance with safety measures and therefore the spread of the virus (Muñiz-Rodríguez et al., 2020; Banerjee et al., 2021).

Several attempts have been made to find pedagogical ways to mitigate this bias. The most straightforward method, i.e., explaining about the bias and the potential perceptual mistakes it can induce, seems to work in certain cases (Lammers et al., 2020), but fails in others (Schonger and Sele, 2020). Other rather simple interventions, such as instructing participants to make estimates through intermediate steps (Lammers et al., 2020) or framing the scenario in terms of doubling times rather than growth rates (Schonger and Sele, 2020) have been shown to significantly reduce the bias. Despite these rather positive findings, other studies did not succeed in attenuating the bias *via* short graphical (Levy and Tasoff, 2016) or other types (using tables or direct non-numerical ways; Wagenaar and Sagaria, 1975; Wagenaar and Timmers, 1979) of interventions.

These mixed results point to the need of understanding better the mechanisms that induce the bias in order to mitigate it more effectively. In the relatively few studies assessing this question, such factors as the level of expertise of the participants (Christandl and Fetchenhauer, 2009) or the manipulation of the relevance of the topic (Romano et al., 2020) seem to have little or no effect on the occurrence of the exponential growth bias. Recently growing attention has been paid to the choice of the scale used in graphical representations of exponential growth (usually, line charts or scatterplots). In particular, as the logarithmic scale makes the exponential curve look linear, it can eliminate the underestimation bias and thus render the graphs more comprehensible (Ciccione et al., 2022). For instance, Hutzler et al. (2021) showed that participants looking at epidemiological data with logarithmically scaled growth curves have made significantly more accurate estimates than those who looked at linearly scaled graphs. In addition to this, with logarithmic scaling, their predictions were not susceptible to range changes on the y-axis as was the case in the linear scale condition, suggesting that participants could compare countries in different phases of infection growth more accurately. Similarly, Ciccione et al. (2022) also identified scaling as one of the factors that can attenuate the misperception of exponential growth when making predictions, alongside the noisiness of data, the task to be performed by the user (pointing vs. guessing a number) and his/her level of mathematical knowledge. However, other studies show that the logarithmic scale induces even stronger exponential growth bias. For instance, Romano et al. (2020) found that when participants are shown exponential growth on a logarithmic scale, they have much more difficulty in describing the graph and making predictions compared to a graph with a linear scale. In a similar vein, Menge et al. (2018) demonstrates that even professional scientists in ecology interpret graphs more accurately when they have linear rather than log-scaled axes.

In the current study, in an experiment with a short educational intervention, we further examine the factors modulating the exponential bias in order to shed more light on the somewhat conflicting results described above and suggest a theoretical explanation for these findings. First, we investigate in more detail the effect of using the linear versus the logarithmic scale in graphs when dealing with exponential growth. Studies on the visualization of other phenomena point out that differences between visualizations of the same data can drastically change the viewer‘s interpretation of information (Padilla et al., 2022). We hypothesize that the contradictory results found in the studies arise from the fact that they test the use of the two scales for different tasks: describing the data in the graph (or simply graph-reading) vs. making predictions on the trajectory of the growth. Specifically, we suggest that when a viewer has to read or describe a graph by attending to the values on the axes and extrapolating them, the linear scale is easier to use, as it can be interpreted straightforwardly, using the habitual tendency to reason linearly (Van Dooren et al., 2007). Indeed, adults with formal Western education tend to map numbers onto space in a linear manner (Dehaene et al., 2008). In this context, the log scale can be difficult to grasp and seem counterintuitive, as steps on a logarithmic scale are not additive but multiplicative (Menge et al., 2018). Several studies have shown that when reading a log-scaled graph, participants with different educational backgrounds confuse the values of the tick marks (Heckler et al., 2013) or tend to make numerical overestimations (Romano et al., 2020; Ciccione et al., 2022). On the other hand, when a person has to make predictions from a graph on the future trajectory of a growth, the log scale seems preferable, as it can help him/her notice the exponentially increasing growth rate even at its beginning, when it can look misleadingly slow on a linear scale (Hutzler et al., 2021). This is especially relevant, when data with differing growth trajectories and/or different orders of magnitude is plotted in the same graph (Perneger et al., 2020). In other words, the overreliance on linearity characteristic to many viewers (Van Dooren et al., 2007) can cause difficulties when using each of the scales in an unsuitable context: on one hand, if the log scale is perceived as linear, there is a risk of misinterpreting the values of the axis in graph description tasks. On the other hand, when the linear scale is used in prediction tasks, viewers might fail to perceive the slope of the growing curve and its exponential trends, leading to less accurate predictions. We investigate this issue by presenting two groups of participants with the same data plotted either on the log, or the linear scale. In both scale conditions, we ask participants the same questions that involve describing the graphs (questions 1–3) and making predictions based on it (questions 4–5). If the exponential bias in graphs is modulated by the presentation of a particular scale in the suitable context, participants in different scale conditions should respond differently to the same questions.

A second factor we examine in this study is the role that mathematical education can have on the perception of exponential growth in graphs. A body of literature demonstrates that mathematical skills and higher levels of numeracy can act as a protective mechanism against cognitive biases and oversimplifications through heuristics (Munoz-Rubke et al., 2022). For instance, higher numeracy was found to be associated with less confirmation bias (Hutmacher et al., 2022), while short educational interventions of mathematical nature were shown to reduce whole-number bias (Thompson et al., 2021). In the context of the exponential growth bias, only two studies directly looked at the effect of mathematical education. While Wagenaar and Sagaria (1975) found that mathematical sophistication of the subjects nor experience with growth processes modulated the bias, Ciccione et al. (2022) showed that higher mathematical knowledge led to smaller underestimation of exponential growth. Nevertheless, these papers assessed only indirectly the level of mathematical education through subjective questionnaires. Other studies on the exponential growth bias just looked at the general education level of their participants (Christandl and Fetchenhauer, 2009; Levy and Tasoff, 2016; Menge et al., 2018). In the current study we tested two groups of undergraduate students who had differing levels of math knowledge due to the nature of their respective curricula. Specifically, one group studied foreign languages and had few basic courses in math at secondary school and no math at university, while the other group studied computer science and had a substantial number of math courses both at secondary school and university. In this way, we ensured that alongside subjective self-evaluations of their math level, we had objective evidence about the education in math that both groups underwent.

For the second part of the experiment, we designed a short educational intervention in order to test if the difficulties of graph interpretation leading to exponential growth bias could be reduced in both scale conditions and across participant groups. Recent papers have called for designing interventions that could increase statistical literacy in general (Gal, 2002; Gould, 2017; Weiland, 2017; Engel, 2021), and the understanding of the exponential bias (Sieroń, 2020; Munoz-Rubke et al., 2022) alongside with the scales used (Menge et al., 2018; Watson and Callingham, 2020; Ciccione et al., 2022) in particular. For each scale condition we came up with short explanations accompanied by graphs that take into account the propositions expressed in several recent studies, including instructions on the organization of the log scale (Ciccione et al., 2022); the presentation and labelling in the graphs (Heckler et al., 2013; Menge et al., 2018); driving the participants’ attention to certain elements of the graph (da Silva et al., 2021) etc.

Finally, following calls to investigate decision making with visualizations in terms of human perception and cognitive theory (Alhadad and Alhadad, 2018), we propose to interpret the results of this study in light of a dual-process model. According to this model, two types of decision-making processes exist: System 1 is used for fast automatic decisions and can be identified with intuitions; while System 2, or reasoning, is used for more rational analytical decisions (Stanovich, 1999; Kahneman and Frederick, 2002; Kahneman and Klein, 2009). This model helps to explain how the human mind deals with the limitations of its processing capacity and, in our view, can shed more light on the causes of the misperceptions arising in graph reading with different scales. We will address this issue in the Discussion section of this paper.

## 2. Part I: Pretest

### 2.1. Methods

#### 2.1.1. Participants

99 participants took part in this online experiment. They were all recruited at Vilnius University and Vilnius Gediminas Technical University. 49 participants were enrolled in a BA degree in foreign languages, while the remaining 50 participants studied computer science. Note that students in computer science were chosen instead of students in mathematics intentionally, as we aimed at testing participants with an intermediate to high level of math, who have had math courses at university and who could represent a more general population with a background in natural/formal science, not just professionals in math. For simplicity, the first group will be labelled in this paper “humanities” and the second “science” group. Participants in each group were randomly assigned to one of the two experimental scale conditions. A between-subject design was chosen in order to avoid possible bias when dealing with both scales at a time. All participants were free to quit the experiment whenever they wanted, thus making sure that only interested and fully engaged participants were completing the experiment. After an initial screening of the data, 4 participants were excluded based on short completion time, resulting in a total of 47 participants in the humanities group, and 48 in the science group.

#### 2.1.2. Stimuli

**Figure 1:**
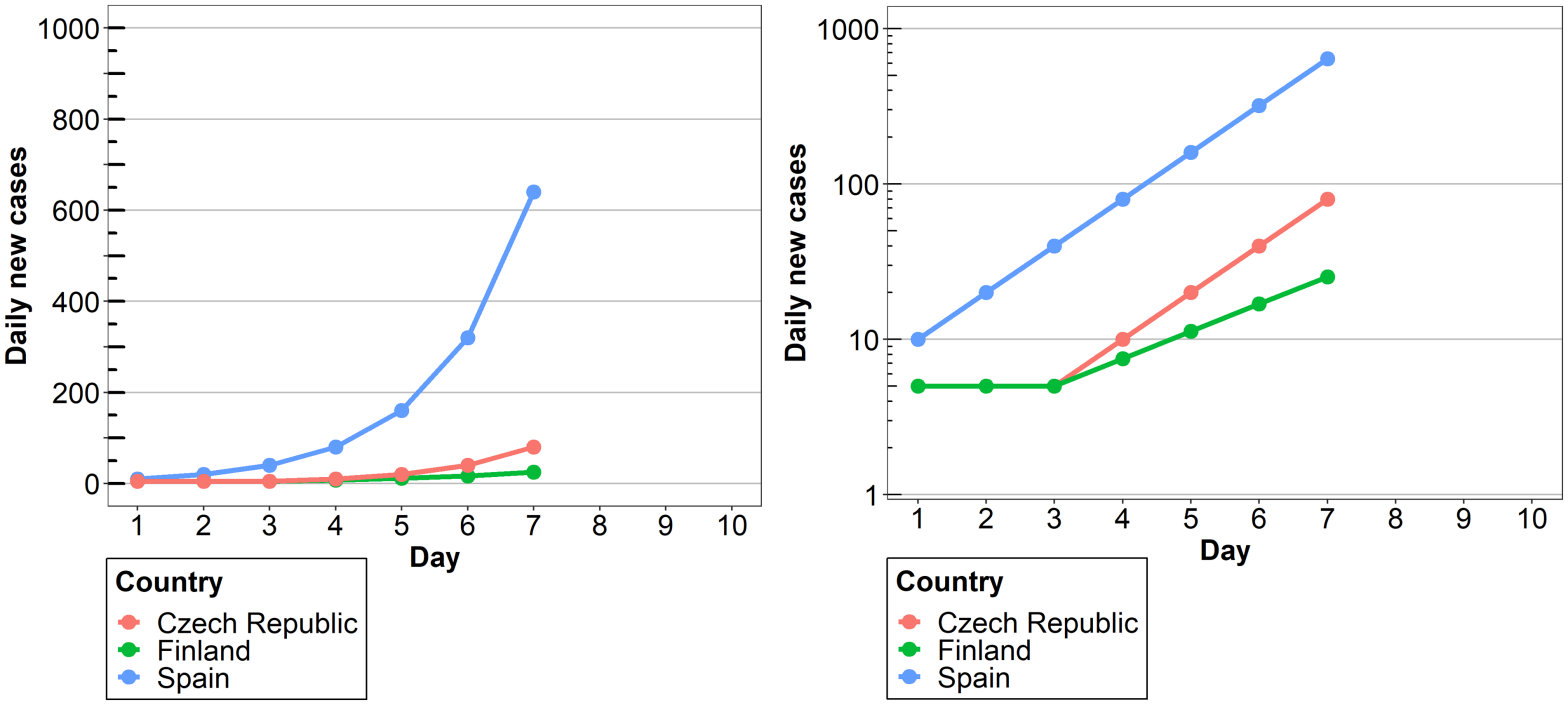
The two graphs presented to participants used in the linear and the log conditions, respectively.

Two line charts for time series representing hypothetical Covid-19 daily case data from 3 countries were designed for the experiment. The Czech Republic, Finland and Spain were chosen for the examples, as they are well known for the Lithuanian participants, but are not associated with specific Covid-19 surges or containment policies, as Italy or Sweden would be. The hypothetical data for the three countries was distributed in such a way as to allow comparisons of large, small and asynchronous outbreaks (Perneger et al., 2020). Namely, Finland represented a smaller outbreak, while the other two showed a larger outbreak that progressed earlier in Spain, compared to the Czech Republic (see Figure 1). The line charts differed only in the scale used for the y axis (representing the daily new cases). Specifically, a linear scale was used for one condition, and a logarithmic scale for the other. For both conditions, only the major labels were shown (0–200–400-600-800-1,000 in the linear scale condition; 1–10–100-1,000 in the log scale condition), as it is common practice in online platform and media coverage across countries (Clement et al., 2020; Idogawa et al., 2020; Wissel et al., 2020). In both conditions the labels went up till 1,000 and were accompanied by grey major gridlines in order to facilitate the readability. In addition to this, minor tick marks without labels were also included in the graphs. This was especially important for the log scale, as there is evidence that in the absence of minor tick marks, people tend to interpret the logarithmic scale as linear (Heckler et al., 2013).

#### 2.1.3. Procedure

Participants in each condition were presented with the corresponding line chart with either the linear or the log scale. In both scale conditions they were then asked the same 5 questions. We came up with three questions testing different aspects of graph description, and two questions assessing prediction-making from graphs. The questions and suggestions on the location (which condition) and the nature of the perceptual errors participants might make are presented in Table 1.

**Table 1:** Questions asked during the experiment and their characteristics.

Question	Question type	Answer type (and proposed answer options)	Expected location and nature of misperceptions
1. Evaluate how many new cases occurred on day 6 in Spain.	Graph description	Ordinal (150; 300; 500; 800)	Log condition: while the correct answer is located halfway between the major tick marks 100 and 1,000, a linear interpretation of the log scale would lead to answering 500 instead of 300.
2. When did the number of daily new cases in Spain increase more?	Graph description	Ordinal (between day 4 and 5; between day 6 and 7; likewise)	Log condition: if participants interpret steps on this scale as linear, they would tend to think that the increase in both periods was identical and would fail to perceive the increasing outburst of cases over time.
3. Look at the difference in daily cases between Spain and the Czech Republic. How did the difference in cases from day 3 to day 7 change?	Graph description	Ordinal (decreased; remained stable; increased)	Log condition: An incorrect interpretation of the log scale would lead to a faulty perception of the growth dynamics of the two lines and the daily increasing difference in cases between them
4. Are cases in the Czech Republic more likely to grow like in Spain or in Finland?	Prediction making	Binary (like in Spain; like in Finland)	Linear condition: at first glance the growth of daily new cases in Finland and the Czech Republic looks more similar than that in Spain due to the differing growth rates between the first two and different growth progressions between the last two. This can lead to choosing the wrong growth trajectory (Finland instead of Spain).
5. What will approximately be the number of new cases in the Czech Republic on day 10?	Prediction making	Continuous (manual entry)	Linear condition: at first glance the growth of daily new cases in Finland and the Czech Republic looks more similar than that in Spain due to the differing growth rates between the first two and different growth progressions between the last two. This might lead the participants to providing a much lower estimate of future growth for cases in the Czech Republic than they actually are.

These questions were followed by two additional questions assessing subjective autoevaluation. Participants were asked to assess their confidence in their answers on a scale from 1 to 5 and to evaluate how difficult the tasks were (scale 1 to 5). At the very end of the experiment (following parts 1 and 2) participants had to indicate their level of math on a scale from 1 to 10.

### 2.2. Results

We use an ordered logistic regression model to analyze the data from the first three questions, as they had ordered responses, which, however, cannot be considered continuous (Long, 1997). These analyses were performed using the polr command from the MASS package in R (Venables and Ripley, 2002). A logistic regression was used to analyze responses from question 4 (binary dependent variable), and a simple linear regression for question 5 (continuous dependent variable). For each of the five questions we constructed a model with Response to question as the dependent variable. Scale condition (linear vs. log) and Group (humanities vs. science), as well as their interaction were included as contrast-coded fixed factors. In questions 1–4, *p*-values were obtained by Wilks’ likelihood ratio tests of the full model against the model without the effect or interaction in question. For question 5, an *Anova* was used for model comparison.

**Figure 2:**
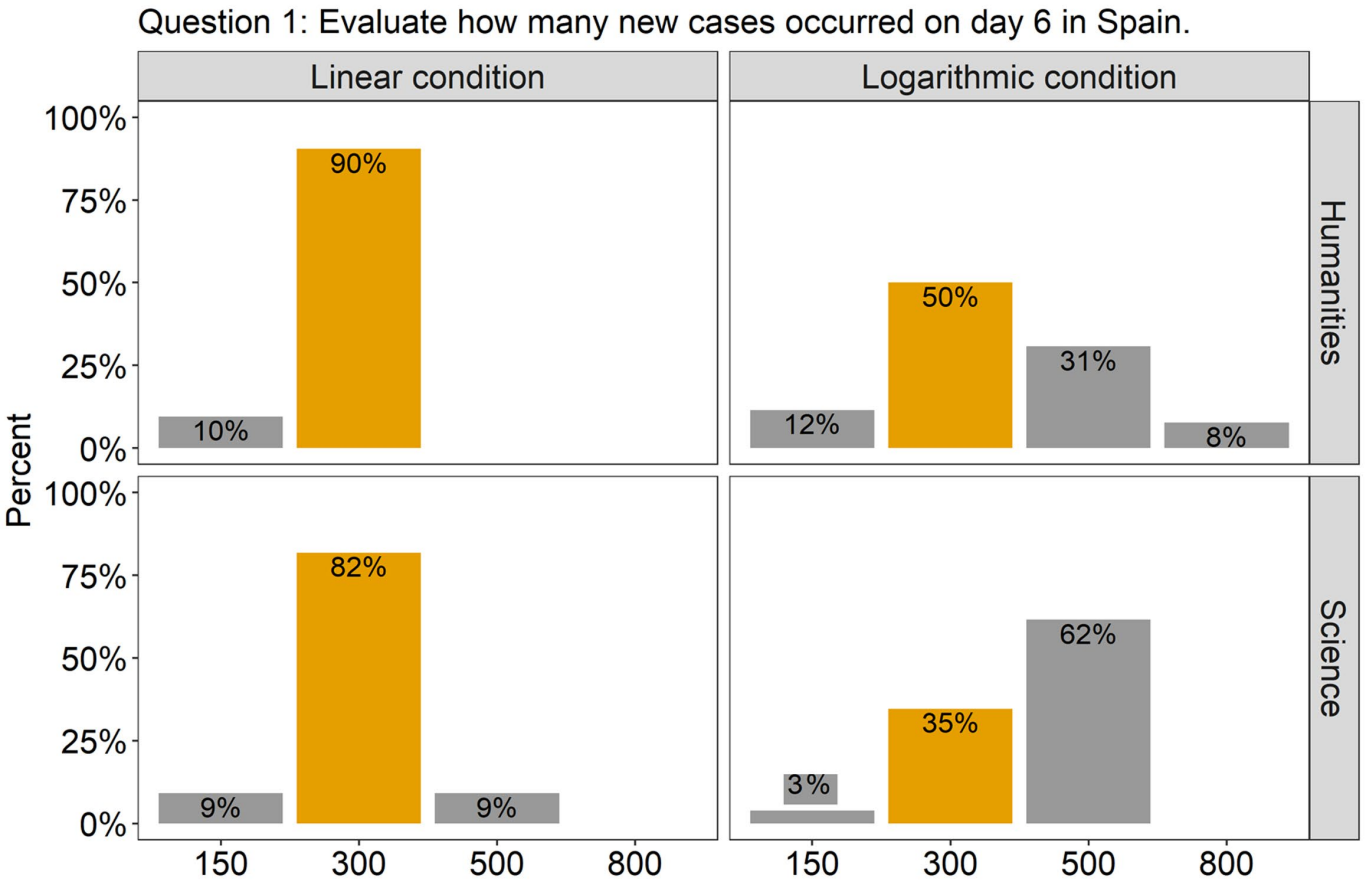
The barplots present the distribution of the participants’ answers to Question 1 (graph description question). The correct answer is indicated with orange-colored bars.

**Figure 3:**
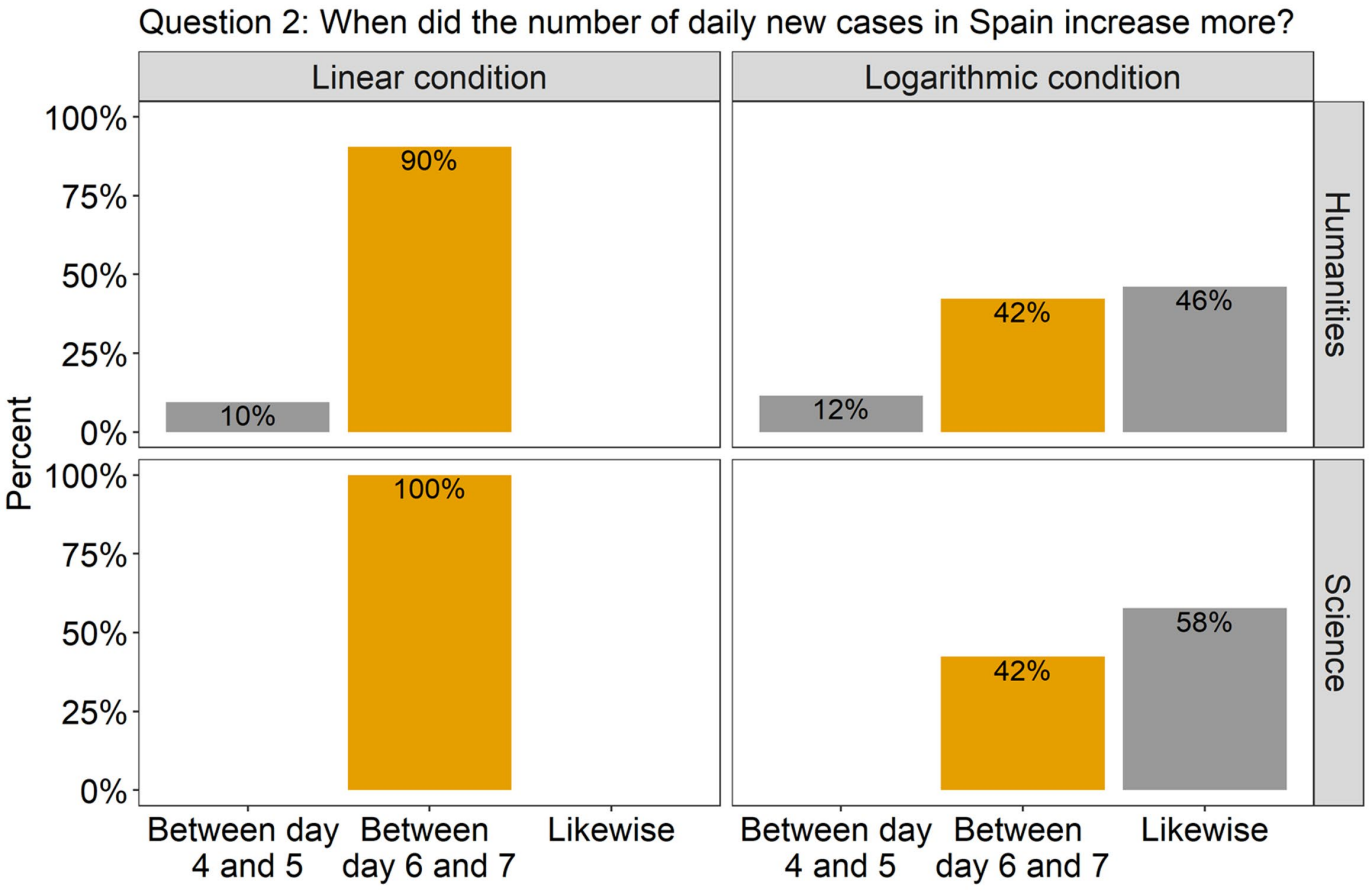
The barplots present the distribution of the participants’ answers to Question 2 (graph description question). The correct answer is indicated with orange-colored bars.

**Figure 4:**
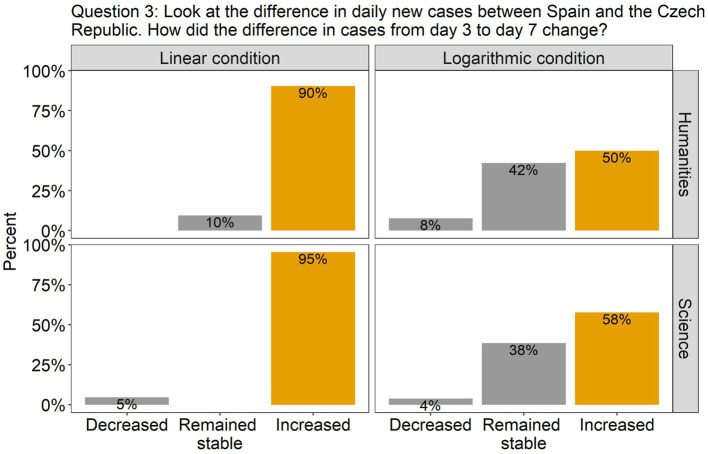
The barplots present the distribution of the participants’ answers to Question 3 (graph description question). The correct answer is indicated with orange-colored bars.

The analysis revealed that for all the questions there was a significant effect of Scale condition, but no effect of Group, nor an interaction between them. Specifically, in question 1, there was a significant difference between the linear and the log conditions [*β* = 2.00, SE = 0.52, χ^2^(1) = 18.51, *p* < 0.0001], with 86% of the participants (across groups) answering accurately in the linear condition compared to only 42% in the log condition (see Figure 2). This indicates that in the log condition many participants wrongly interpreted the values of the intermediate tick marks. As the target point was located halfway between the tick marks 100 and 1,000, they estimated that the value on the y axis was 500, instead of 300. Interestingly, although the difference between groups was not significant, we can see from the graph that many more participants from the science group made this mistake compared to the humanities group (62% vs. 31%). Turning to question 2, there were more than twice as many correct answers in the linear scale condition (95%) compared to the log scale condition (42%) [*β* = 2.79, SE = 0.67, χ^2^(1) = 27.22, *p* < 0.0001; see Figure 3]. Importantly, in the log condition a striking 52% of the participants clearly misunderstood the log scale in terms of a linear scale. When describing changes on this single curve, they misunderstood the pattern of change between two consecutive points: they thought that distances between points on the y axis are the same, independently on their location. A similar tendency can be observed in question 3 (see Figure 4), where the difference between scale conditions [*β* = −2.45, SE = 0.69, χ^2^(1) = 19.38, *p* < 0.0001] was due to the great majority of participants (93%) answering correctly in the linear condition as opposed to 54% in the log one. This suggests that in the log condition participants misperceived the increasing distance between the two curves.

**Figure 5:**
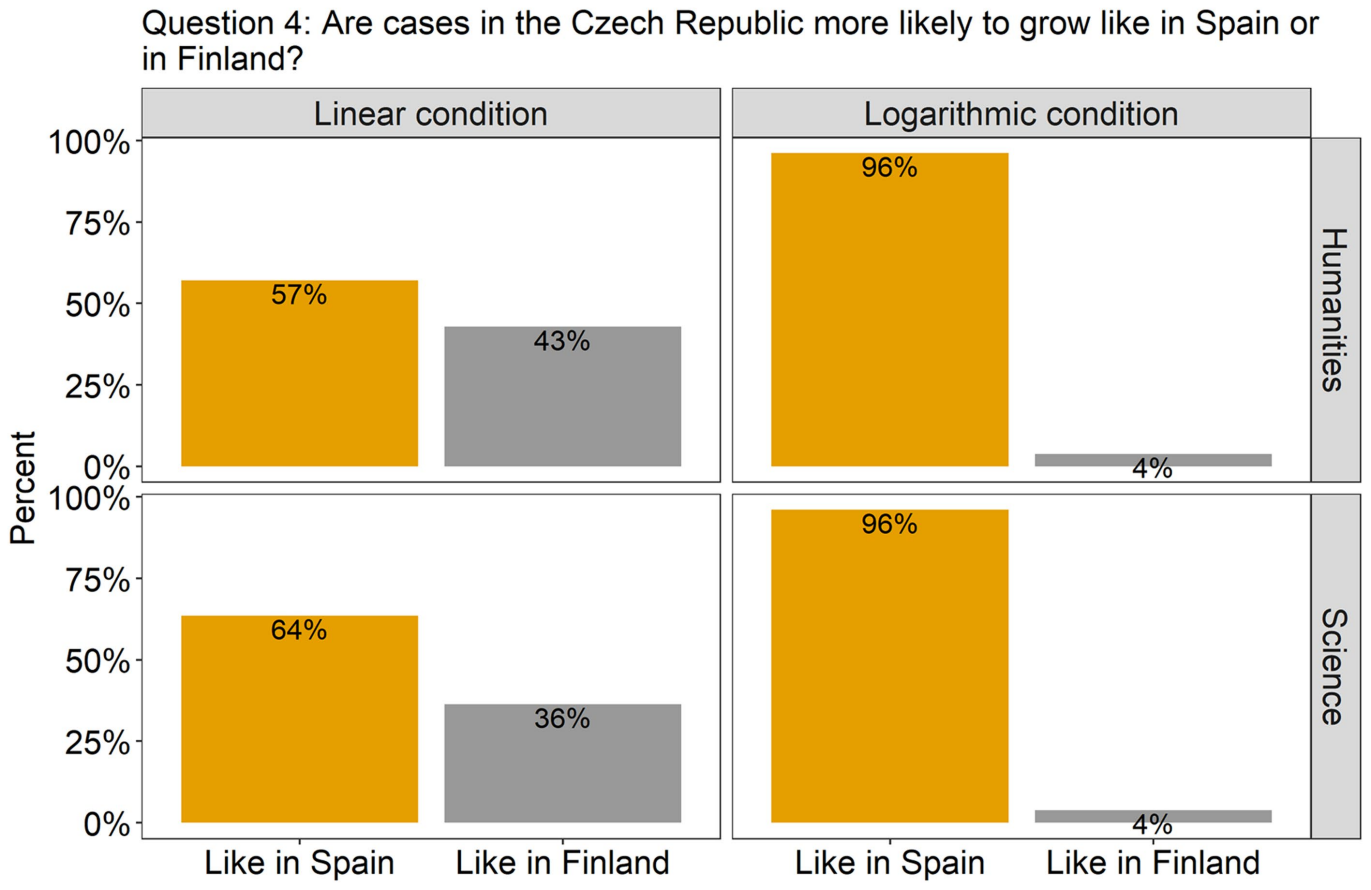
The plots present the distribution of the participants’ answers to question 4 (prediction). The correct answer is indicated with orange-colored bars.

**Figure 6:**
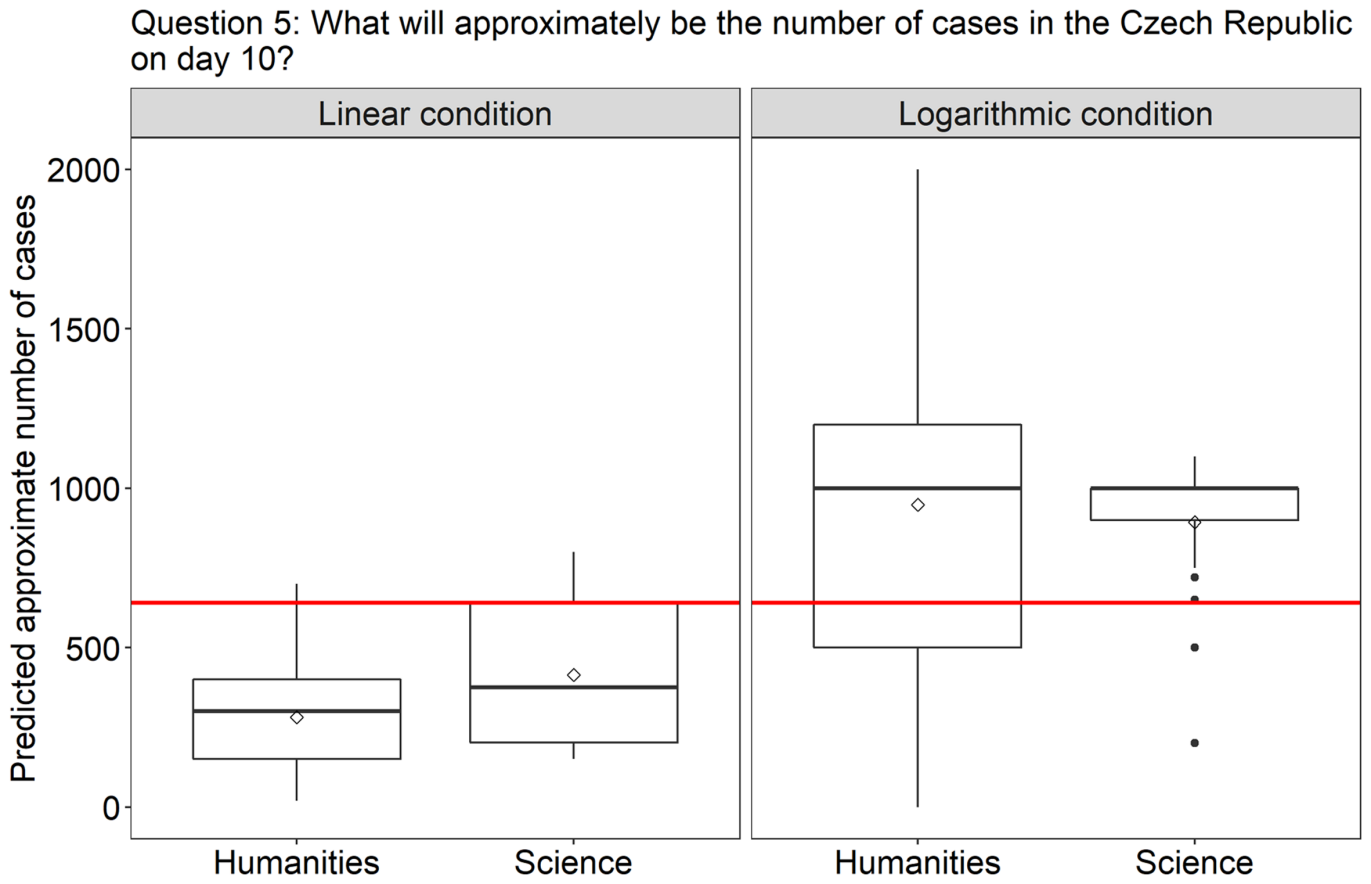
The plots present the distribution of the participants’ answers to question 5 (prediction). The correct answer is indicated with a red line.

Turning to the questions involving predictions, in question 4 (Figure 5), the effect of Scale condition was again significant [*β* = 2.80, SE = 0.79, χ^2^(1) = 20.39, *p* < 0.0001], but this time the participants were much more accurate in the log scale condition (96% answered correctly) than in the linear scale condition (only 60% answered correctly). Finally, in question 5, where participants had to estimate the approximate number of new cases in the Czech Republic on day 10, a difference between the scale conditions was also observed [*β* = 756.23, SE = 163.71, *F*(1, 91) = 21.34, *p* < 0.0001]. As can be seen from Figure 6, participants in both groups underestimated the growth in the linear scale condition, but overestimated it in the log condition. There was homogeneity of variances, as assessed by the Levene’s test for equality of variances, for both Group (*p* = 0.06) and Scale condition (*p* = 0.2).

Finally, we carried out t-tests to examine whether the participants’ answers on the additional questions differed depending on their educational background. First, we found that participants from the science group reported significantly higher scores on math autoevaluation than participants from the humanities group [on a scale from 1 to 10: science: mean = 6.94; humanities: mean = 5.06; *t*(91.79) = −4.79, *p* < 0.0001]. Next, we looked at the difference between groups in their level of confidence. Here too the difference was significant, namely, participants with a background in science felt more confident in their answers [on a scale from 1 to 5: science: mean = 3.56; humanities: mean = 3.02; *t*(90.75) = −2.98, *p* < 0.01]. Finally, the analyses revealed that participants in science found the tasks to be easier than their peers in humanities did [on a scale from 1 to 5: science: mean = 1.42; humanities: mean = 1.85; *t*(91.31) = −2.29, *p* = 0.02].

### 2.3. Discussion

The choice of the scale impacts indeed the responses of the participants. However, it is not the case that one scale is overall better than the other. Rather, each of the scales can induce errors in a particular context. Similarly to previous studies (Heckler et al., 2013), our experiment has shown once more that people misunderstand the minor tick marks on the log scale, and instead process them in terms of a linear scale. For this reason, the complexity of the log scale resulted in an inability to use it effectively to describe data on a graph. On the other hand, it proved to be very helpful in making predictions about future growth. Conversely, the experiment showed that the linear scale is much easier to use when describing a graph. Note, that participants in the linear scale condition reached very high accuracy (around 90% correct) on the first three questions. Nevertheless, this tendency was reversed in question 4, where participants had to compare the curves and predict their future growth. In this case, around 40% of the participants chose the wrong answer.

Interesting results were obtained on question 5. Here, participants in both conditions gave slightly inaccurate responses, but the nature of their mistakes was diametrically opposed. In particular, they underestimated the growth in the linear, but overestimated it in the log scale condition. Concerning the linear condition, this tendency reflects the typical exponential growth bias found in a variety of studies (Wagenaar and Sagaria, 1975; Hutzler et al., 2021). The tendency found in the log scale condition can seem more surprising, but it has already been observed by two other studies (Romano et al., 2020; Ciccione et al., 2022). The latter study found that the overestimation effect occurred when participants were presented with a noiseless exponential function rather than noisy data, which was also the case in our theoretical data scenarios. The overestimation effect found in the log scale condition could be overall considered preferable to the underestimation bias in many contexts. For instance, in cases of epidemics, the mere detection of exponential growth *per se* matters, while the exact estimate of the final numbers is not indispensable (Hutzler et al., 2021). On the other hand, these findings suggest that the choice of the scale could be motivated by the message one would wish to convey. Specifically, the log scale could be used in order to stress the importance of the growth of a phenomenon, while the linear scale could help to downplay its gravity. Note, however, that these tactics could also be employed to manipulate the viewer, and therefore a better understanding of these perceptual effects in the general public would be preferable.

Turning to the second factor we examined in this study, mathematical education does not seem to play a major role in the perception of exponential bias. Specifically, independently on their background in humanities or in science, both groups of participants were susceptible to the exponential growth bias when interpreting graphs plotted with an inappropriate scale. Yet, we found that participants in science group reported significantly higher autoevaluation in math levels, they felt more confident in their answers and had lower scores on perceived difficulty of the tasks. This points to one of the causes for the persistence of this bias: people are simply not aware of their lack of understanding of exponential growth. Christandl and Fetchenhauer (2009) also found that people are overconfident in their capacity to solve problems that involve exponential growth, which results in a low demand for corrective tools.

## 3. Part 2: Intervention and posttest

In order to test whether the difficulties experienced when using the log scale in describing a graph, and the linear scale when making predictions can be overcome, we designed a short intervention, which will be described next.

### 3.1. Methods

#### 3.1.1. Participants

The same participants were tested as in part 1.

#### 3.1.2. Stimuli

For each of the scale conditions we designed a short intervention consisting of two-slides-long instructions with graphs. We presented the same data as in part 1, but with additional information on the graphs and/or in the description, based on cumulated recommendations from several previous studies. For the log condition, we encouraged the participants to examine the y axis, and explained briefly the principles behind the log scale. We first explicitly drew their attention to the major tick marks and explained that each label is 10 times as large as the previous one (i.e.,1 > 10 > 100 > 1,000). We then provided information about the uneven distribution of the minor tick marks. In order to facilitate the understanding of this concept, we added a higher density of numerical labels in between major ticks (Heckler et al., 2013; Ciccione et al., 2022). In addition to this, we included additional gridlines at each minor tickmark.

In the linear condition we also added intermediate tick labels and gridlines. Moreover, we emphasized the fact that outbreaks can differ in their size and/or their timing. We encouraged participants to compare the three lines in terms of this information. Similar instructions that encourage the noticing of particular elements in the graph have been shown to help the viewers (Chang et al., 2016; Boone et al., 2018). The materials used for the educational intervention can be found in Supplementary materials.

#### 3.1.3. Procedure

Participants were first presented with the educational intervention consisting of two slides with graphs and instructions. Following these slides, they saw the modified graphs with the same data and were asked to answer again the same questions. Participants were told that they either could answer as in the pretest, or modify their answers if needed. They were then asked to evaluate how useful the intervention was. Finally, participants were asked basic demographic questions (age, studies, gender) and to evaluate their level in math.

### 3.2. Results

As we have already shown in part 1 each of the scales can cause difficulties in a specific context. Therefore, results from the intervention will be presented by scale condition for those specific difficult questions, namely, the graph description questions for the log scale, and the prediction questions for the linear scale.

For the log condition, we looked at the first three questions (i.e., description of the graph) and we used an ordered logistic regression model to analyze the data. For each of them, we constructed a model with Response to question as the dependent variable. Session (pretest vs. posttest) and Group (humanities vs. science), as well as their interaction were included as contrast-coded fixed factors. *p*-values were obtained by likelihood ratio tests of the full model against the model without the effect or interaction in question. A summary of main effects and interactions that turned out to be significant in both parts of the experiments is presented in Table 2. The figures presenting the results for all five questions of the posttest can be found in Supplementary materials. For question 1 we found a significant effect of Session [*β* = 1.04, SE = 0.41, χ^2^(1) = 6.63, *p* < 0.01] and an interaction between Session and Group [*β* = −2.11, SE = 0.82, χ^2^(1) = 6.87, *p* < 0.01]. *Post-hoc* analyses revealed that the interaction was due to the fact that the difference between sessions was significant in the science group [*β* = −3.02, SE = 0.84, χ^2^(1) = 51.68, *p* < 0.001], but not in humanities (*p* > 0.05). That is, while in science group the accuracy improved from 35% to 89%, it only raised from 50% to 58% in the humanities group. Thus, there was a learning effect following the intervention in the former group, but not in the later. Turning to question 2, there was a significant effect of Session [*β* = −1.57, SE = 0.45, χ^2^(1) = 13.24, *p* < 0.001]. Specifically, in both study groups the correct answer was chosen only 42% of the times at pretest. At posttest, however, the accuracy improved in both groups, raising to 73% and 96% of correct responses in humanities and in science groups, respectively. Although the effect of Group was not significant, nor was the interaction, we still can note that the participants in science group benefited more from the intervention, almost reaching a ceiling effect at posttest. In question 3, we found significant effects of Session [*β* = 0.94, SE = 0.45, χ^2^(1) = 4.62, *p* < 0.05] and Studies [*β* = 1.08, SE = 0.45, χ^2^(1) = 6.17, *p* < 0.05]. Although the interaction only marginally approached significance (*p* = 0.06), we can observe a much stronger improvement in the science group following the intervention (the correct response rate raised from 58% to 89%, compared to 50% vs. 54% in the humanities group).

**Table 2:** Summary of the main effects and interactions that turned out to be significant in both parts of the experiment.

Question	1	2	3	4	5
Part 1: Pretest	Scale condition	Scale condition	Scale condition	Scale condition	Scale condition
Part 2: Posttest	Session Session × Group	Session	Session Group	Session	Session Group

Turning to the linear scale condition and the questions involving predictions, a logistic regression was used to analyze responses from question 4, and a simple linear regression for question 5. Here too, Session (pretest vs. posttest) and Group (humanities vs. science), as well as their interaction were included as contrast-coded fixed factors. A significant effect of Session was found for question 4 [*β* = −2.22, SE = 0.71, χ^2^(1) = 14.02, *p* < 0.001]. Both groups showed similar levels of improvement, on average from 61% at pretest to 93% at posttest.

Finally, for question 5, both the factors Session [*β* = 162.36, SE = 47.39, *F*(1, 82) = 11.74, *p* < 0.001] and Studies [*β* = 170.74, SE = 47.39, *F*(1,81) = 12.98, *p* < 0.001] turned out to be significant. While both groups showed improvement after the intervention by reducing the underestimation tendency, this effect was much stronger in the science group (the correct answer to question 5 was 640; the mean predicted value in humanities at pretest was 282 and 406 at posttest; while in science it was 414 at pretest and 615 at posttest).

### 3.3. Discussion

The results of part 2 of the experiment show that even a short educational intervention can improve the reading and interpretation of graphs involving exponential growth bias. Specifically, it proved to be helpful in dealing with graphically presented data, under conditions when the use of the log and the linear scales causes the most mistakes (i.e., the log scale for the description of a graph and the linear scale for predictions). This result is important as in present times far-reaching measures related to crucial issues such as economic crises and hyperinflation or outbreaks of infectious deceases, such as Covid-19, are often explained with the help of data visualizations, while the general population has low levels of statistical literacy (Bakker and Wagner, 2020). As providing full-scale courses in statistics would be hardly possible for obvious reasons, the effectiveness of such short interventions is encouraging.

Nevertheless, we found a difference between groups in the majority of questions, that did not occur at pretest. Namely, participants in the science group seemed to benefit more from the intervention and showed a greater learning effect. This suggests that the intervention could potentially be adapted to different groups in order to maximize its effectiveness. The possible causes of the difference observed between groups at posttest are discussed in the following section.

## 4. General discussion

The current study demonstrated that the choice of the scale used to represent exponential growth in graphs can have a dramatic effect on the interpretation of these visualizations. The results confirmed our hypothesis that one scale is not overall better than the other. Rather, each of them can cause difficulties in a specific context. In particular, while the log scale leads to more errors when describing a graph, the linear scale can mislead people when they have to make predictions on the future trajectory of exponential growth. This at least partly explains why different studies obtained conflicting results as to which scale is more difficult to use. The second part of the study revealed that these difficulties with both scales can be reduced by means of a short educational intervention. Interestingly, while in the first part (pretest) there was no difference between participants with a background in science and those with a background in humanities, this difference was observed in the posttest. In particular, although both groups benefited to a certain extent from the intervention, the learning effect was much stronger in the science group.

We propose that our findings can be interpreted in light of a dual-process model. In particular, according to this model, reasonably accurate and effective decisions provided by System 1 are sufficient for the hundreds of decisions one has to make on a daily basis, although they might be prone to some errors (Stanovich, 1999). However, in situations where the mental shortcuts are not available and/or high levels of accuracy are required, the effortful System 2 comes at hand. In the field of visual processing, research has demonstrated that a limited set of visual features are detected preattentively (Healey and Enns, 2012). According to Padilla et al. (2018), who proposed an integrated model of decision making with visualizations, decisions based on graphs can be made by using either System 1 or System 2 processing. In the first scenario, viewers unconsciously focus on the aforementioned salient features and use minimal working-memory capacity, while in the second one they employ top-down attentional search of the visual array, which is taxing working memory, but might be more accurate.

In light of this theory, our results could be interpreted in the following manner: when describing graphs (question 1–3) in the linear condition, the viewers could rely on the salient graphical features, such as slopes, and automatically extract the necessary visual information. In other words, they could answer the questions in one or two steps, without having to analytically examine the different elements of the visualization, thus engaging little working memory. In this case, the use of System 1 was sufficient to provide accurate answers to the questions. On the contrary, more complex reasoning and more steps had to be involved in the log condition for the same questions. In particular, the reading and interpretation of the log scale *per se* required more attentional resources (i.e., driving one’s attention to the scale of the y-axis, extrapolating the values of the major ticks, then the minor ticks, etc.). The resulting difficulty of participants to interpret the graph in this condition points to a persistent use of System 1 instead of the required System 2. In particular, it is likely that the viewers used heuristics usually employed to view graphs on the linear scale, which turned out to be misleading in this context.

Conversely, in the prediction question 4, participants could provide effortless accurate answers in the log condition, as it required only minimal reading and interpretation effort (they only had to look at the slopes of the curves and mentally prolongate them, as here the reading of the scale was not necessary to provide the correct answer). On the contrary, in order to be able to answer these questions in the linear condition, one would have to resort to graph analysis and inference making (compare the growth rate of all lines, evaluate their level of progression and synchronicity etc.). In question 5, where more analysis and the use of system 2 was necessary in both scale conditions (in both cases the viewers had to identify the 10th day on the x axis, then decide on where the line must continue, mentally draw it, after that extrapolate a number from the scale on the y axis etc.), many participants still applied linear thinking which turned out to be inadequate for the task. This resulted in underestimation in the linear condition and in overestimation in the log condition.

Thus, the results of the present study suggest that when dealing with graphs representing the exponential growth, viewers rely on salient features without examining the graph analytically and tend to use heuristics characteristic to System 1 processing. These involuntary shifts in focus to salient features bias the perception of graphs and can be detrimental to decision making (Padilla et al., 2018). Unfortunately, we did not record reaction times, which could provide further support for this interpretation of the results. The inclusion of reaction times along with accuracy scores would allow future studies to examine in more detail the possibility of using a computationally high System 2 for more difficult graph reading and prediction-making tasks.

Our second finding that the intervention overall improved the performance of both participant groups might also be explained in light of dual-processing theories. Specifically, it could be the case that both groups made mistakes at pretest as they tried to answer the questions intuitively by using System 1 in tasks which required the application of System 2. However, the instructions and information provided in the educational intervention pushed participants to deliberately pay more attention to certain elements of the graphs and to examine them analytically, thus employing System 2. This resulted in an overall better performance at posttest across groups. This raises the question whether the intervention was effective due to its pedagogical content, or rather it acted as a trigger to switch to a more analytical mode of processing. A future study could address this question by comparing the effect of two interventions, one containing pedagogical content, another – simple instructions to pay more attention to the different parts of the graph.

Turning to the difference found between groups at posttest, several explanations could account for a higher learning effect in participants with a background in science. For example, it is likely that the knowledge and skills they ought to have acquired during their studies got activated following the intervention. Alternatively, it could be the case that these participants were more receptive to the educational contents of the intervention. Previously acquired mathematical knowledge has been found to improve the overall capacities in conditional reasoning (Lehman and Nisbett, 1990; Gillard et al., 2009; Toplak et al., 2012). This entails that students who took a relatively large number of courses in math were more likely to employ strenuous System 2 processing (Borodin, 2016).

Note, however, that even if this was the case, the comparable performance of both groups at pretest on difficult questions point to a persistent use of System 1 processing. This suggests that when viewing graphs which use the inappropriate scale to represent exponential growth in the data, even the relatively “trained” viewers do encounter problems. For this reason, it is crucial for graph design to choose the scale that would direct participants’ attention to the most important information. This would allow them to accurately and effortlessly extract the necessary information without having to resort to System 2. As pointed by Card et al. (1999) visualizations should capitalize on those visual biases which are consistent with the correct interpretation of the data. In our case, this would mean using the linear scale for the description of graphs representing exponential growth, and using the log scale to emphasize the growth when predictions have to be made. This is especially relevant when graphs are used to convey important information to the general public (Bakker and Wagner, 2020).

Finally, it is likely that the performance of the viewers, irrespective of their background, could be improved by teaching them general principles of graph reading. In particular, these skills are not necessarily directly trained in traditional math courses, thus even viewers with a background in science might benefit from such training (Bakker and Wagner, 2020). At the same time, it would not require specific mathematical knowledge, and thus would be easily applicable in curricula in various fields. For instance, da Silva et al. (2021) propose that the viewer should be encouraged to engage in four levels of graph reading and interpretation (i.e., reading, interpretation, prediction making and critical assessment) in order to develop a habit to examine graphs analytically and thus improve their accuracy. Overall, it is equally important to both avoid common pitfalls when designing graphs, as well as to improve the skills of the viewers by means of educational interventions. This is in line with the numerous calls to improve the didactics of many mathematical and statistical topics, as well as statistical literacy and graph-reading skills in the general population, which became crucial in our modern data-driven society (Gal, 2002; Sharma, 2017; Bakker and Wagner, 2020; Watson and Callingham, 2020).

## Data availability statement

The datasets presented in this study can be found in online repositories. The names of the repository/repositories and accession number(s) can be found at: https://midas.lt:443/action/resources/836f4caf-d7a0-480b-aa7a-5d5e0b1c6b90.

## Ethics statement

Ethical review and approval were not required for the study on human participants in accordance with the local legislation and institutional requirements. Written informed consent for participation was not required for this study in accordance with the national legislation and the institutional requirements.

## Author contributions

GM-L, VT, and GinD contributed to grant application. All authors contributed to conception and design of the study. LA and AU conducted the study and collected the data. GM-L performed the data analysis. All authors contributed to the article and approved the submitted version.

## Funding

This project has received funding from European Social Fund (project No 13.1.1-LMT-K-718-05-0034) under grant agreement with the Research Council of Lithuania (LMTLT).

## Conflict of interest

The authors declare that the research was conducted in the absence of any commercial or financial relationships that could be construed as a potential conflict of interest.

## Publisher’s note

All claims expressed in this article are solely those of the authors and do not necessarily represent those of their affiliated organizations, or those of the publisher, the editors and the reviewers. Any product that may be evaluated in this article, or claim that may be made by its manufacturer, is not guaranteed or endorsed by the publisher.
