# Supplementary material for: Is my visualization better than yours? Analyzing factors modulating exponential growth bias in graphs
Source: Front Psychol. 2023 Feb 16;14:1125810. doi: 10.3389/fpsyg.2023.1125810 (PMC9977824; doi:10.3389/fpsyg.2023.1125810)

Supplementary Materials

1 Figures from Part 2 of the study, showing the participants' performance following the educational intervention.

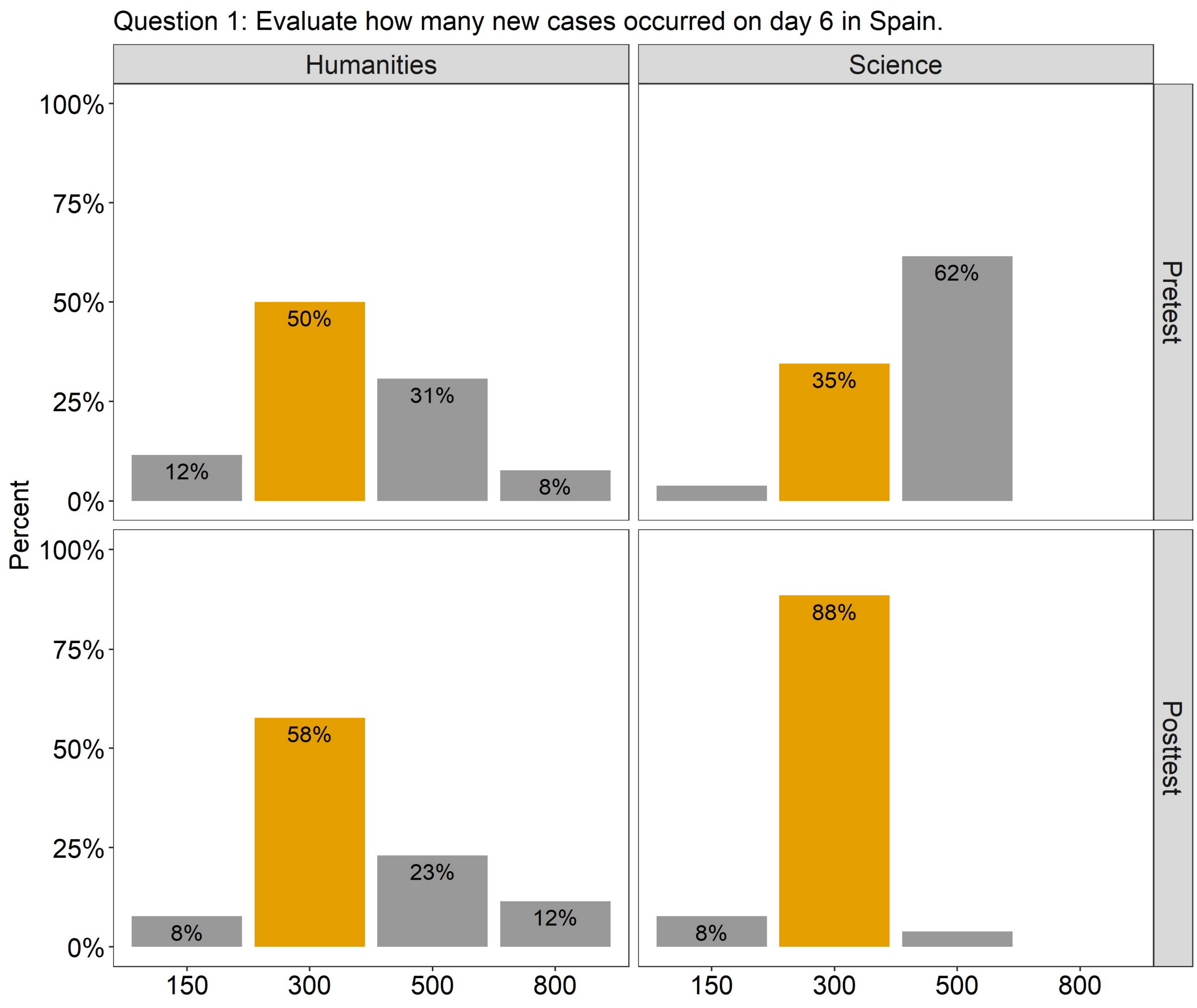

The barplots present the distribution of the participants' answers to Question 1 (graph description question). The correct answer is indicated with orange-colored bars.

Question 2: When did the number of daily new cases in Spain increase more?

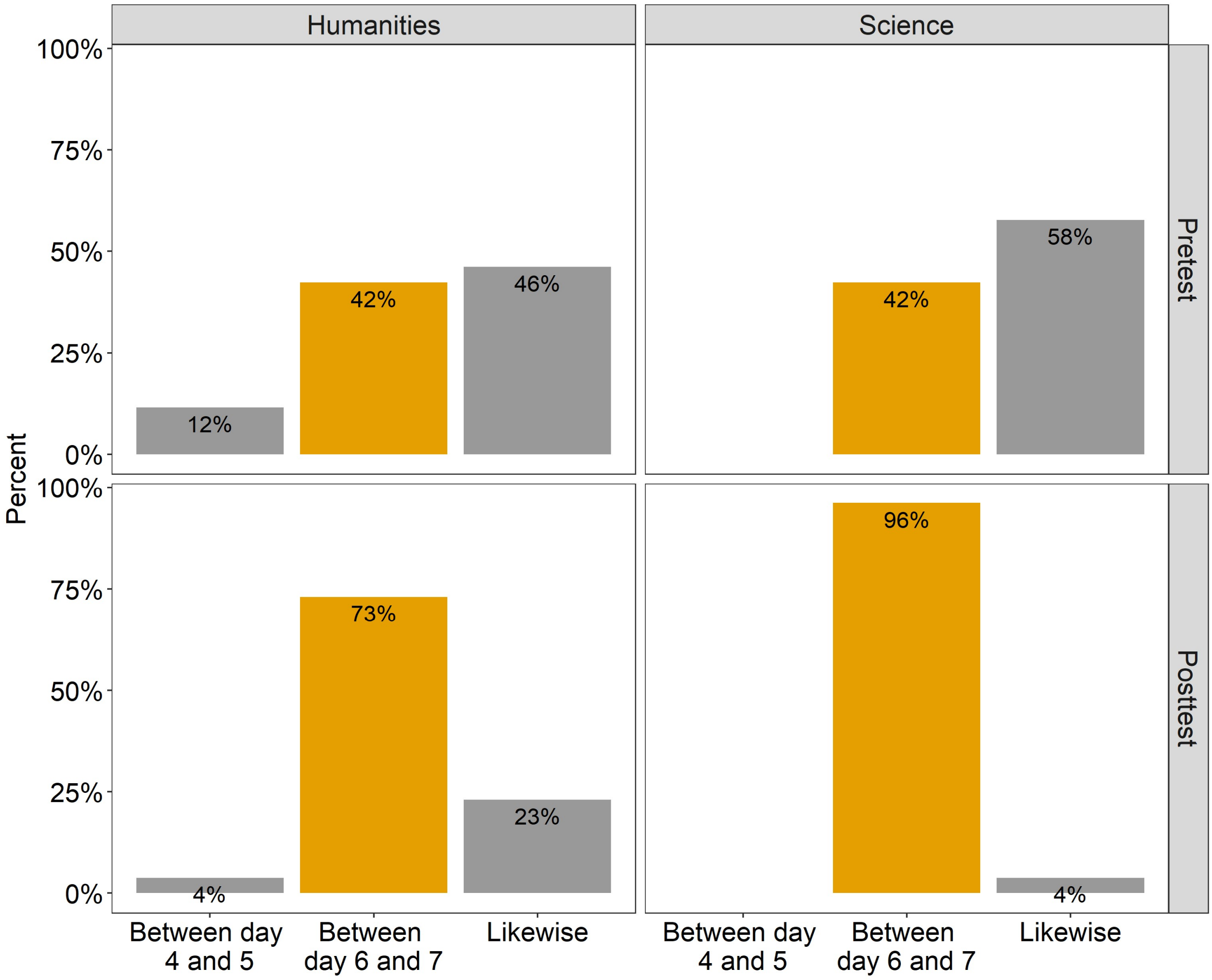

The barplots present the distribution of the participants' answers to Question 2 (graph description question). The correct answer is indicated with orange-colored bars.

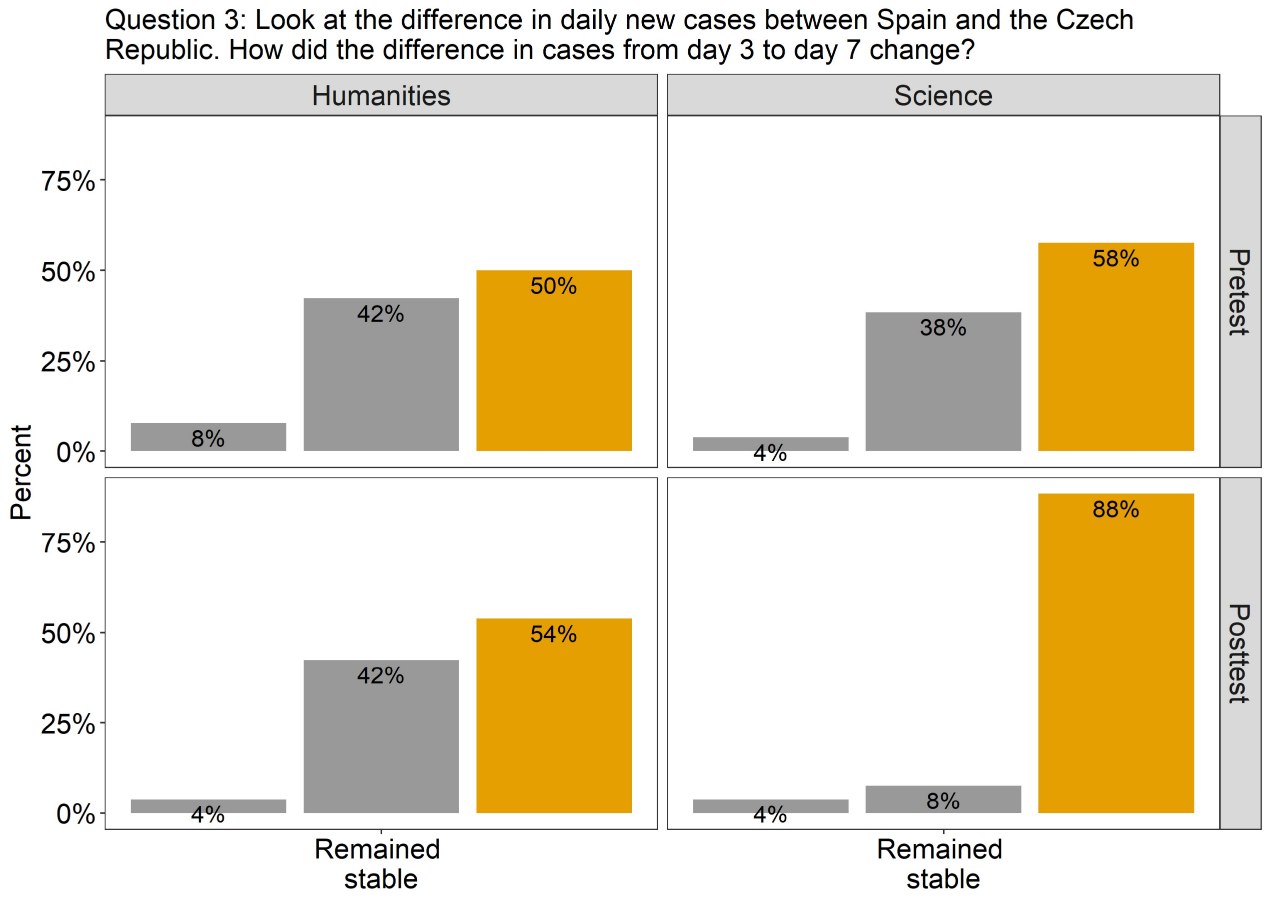

The barplots present the distribution of the participants' answers to Question 3 (graph description question). The correct answer is indicated with orange-colored bars.

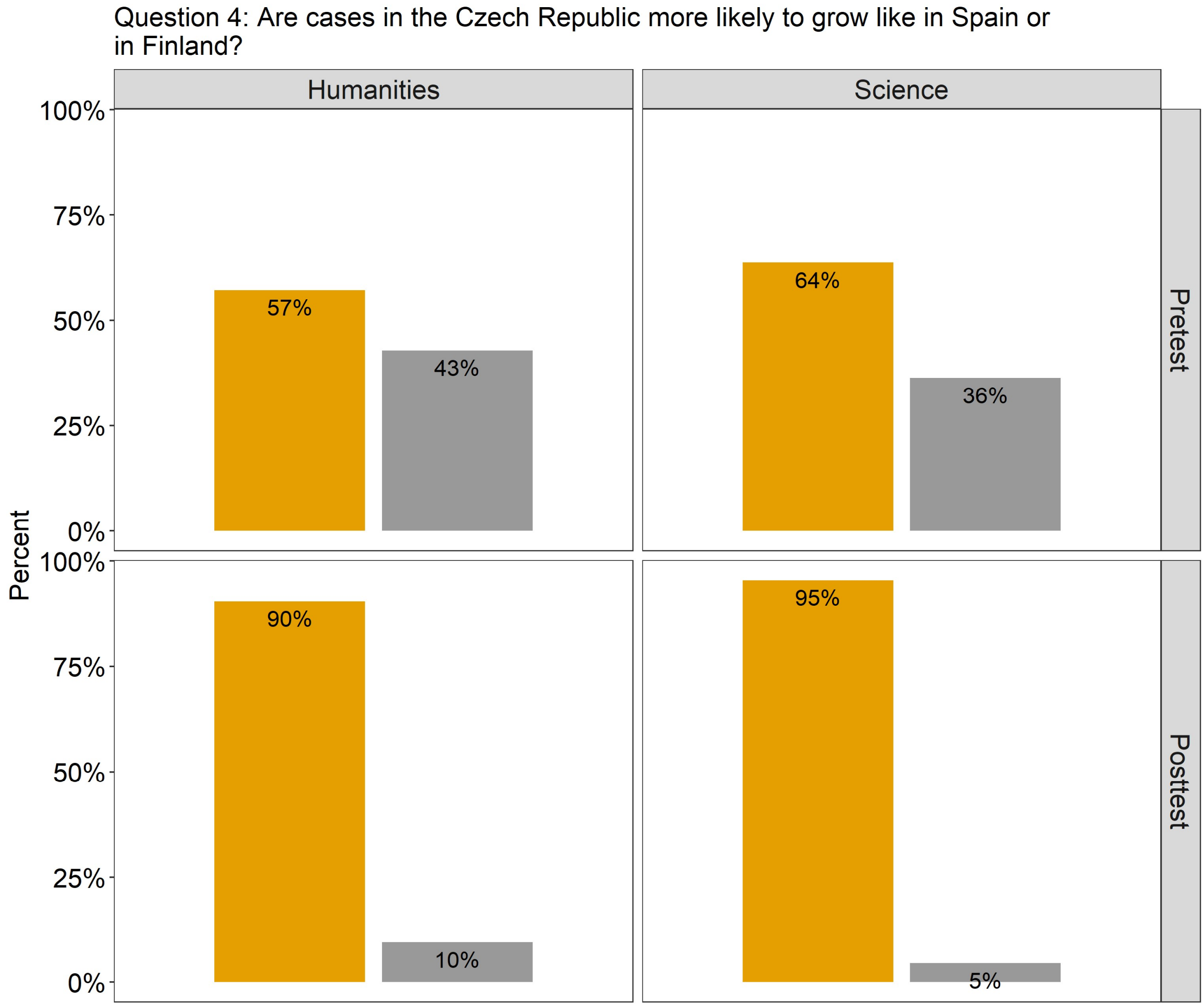

The barplots present the distribution of the participants' answers to Question 4 (prediction). The correct answer is indicated with orange-colored bars.

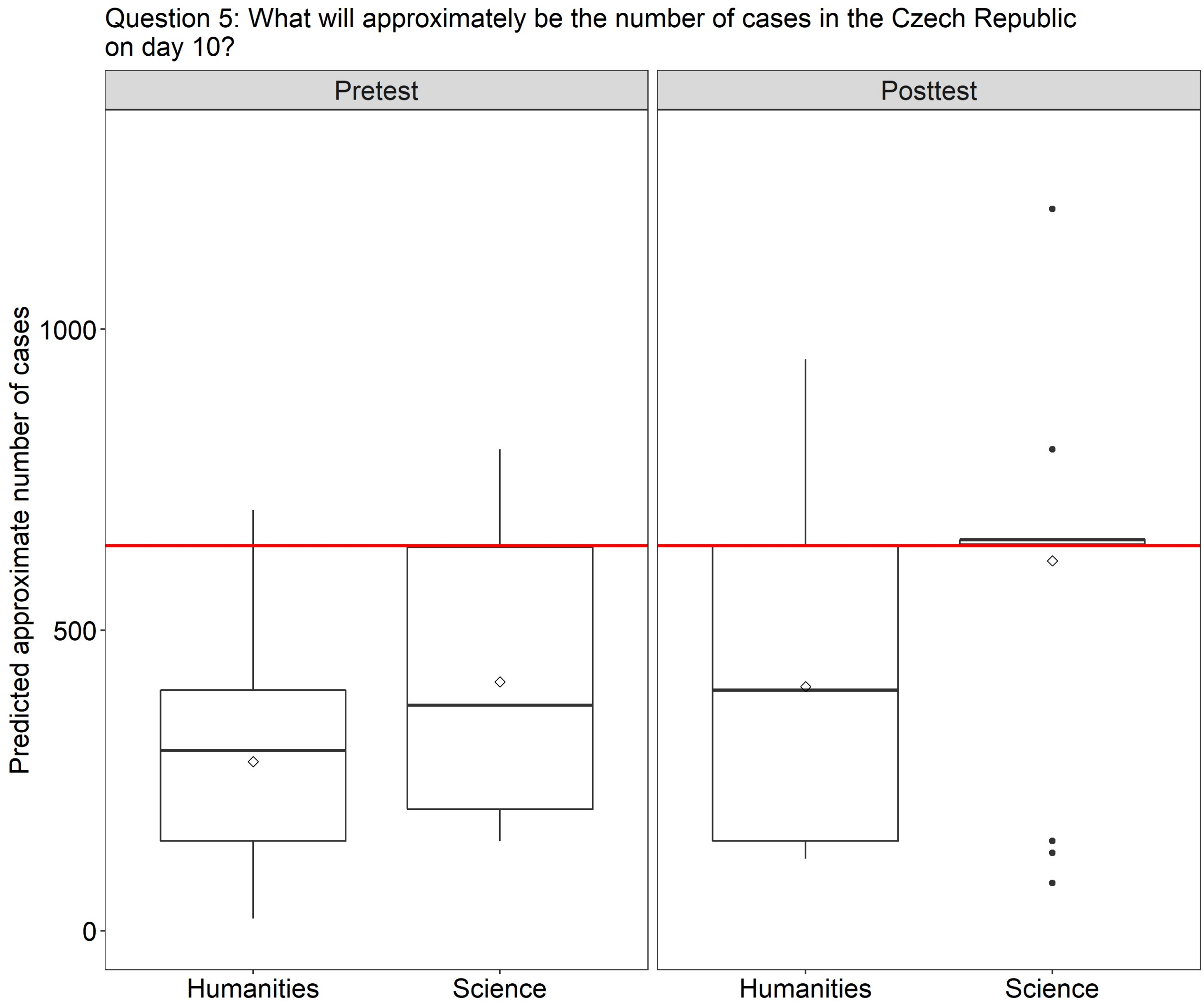

The boxplots present the distribution of the participants' answers to Question 5 (prediction). The correct answer is indicated with a red line.

2 Educational Intervention

2.1 Log scale condition (translated from Lithuanian).

Note that the y-axis (vertical axis) uses a logarithmic scale. This means that the value at each major tick mark is multiplied by 10 each time. These tick marks are distributed evenly troughout the axis (the distance between two tick marks is the same).

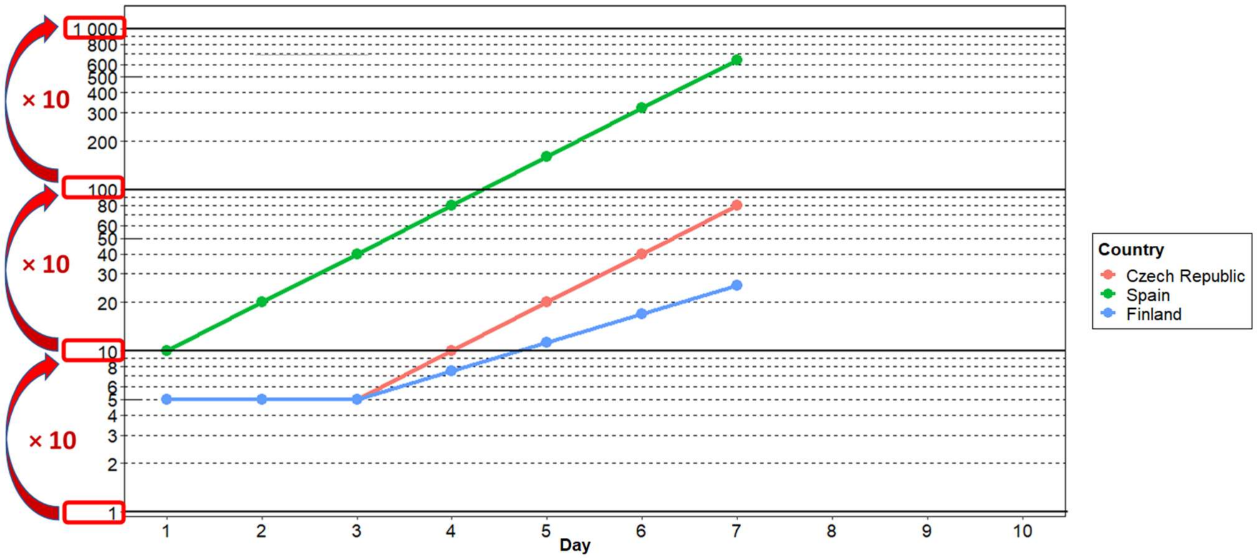

While the minor tick marks are distributed unevenly. Note that the distance between 1 and 2, between 2 and 4, and between 4 and 8 are of equal length, because these pairs differ by the same multiplier 2.

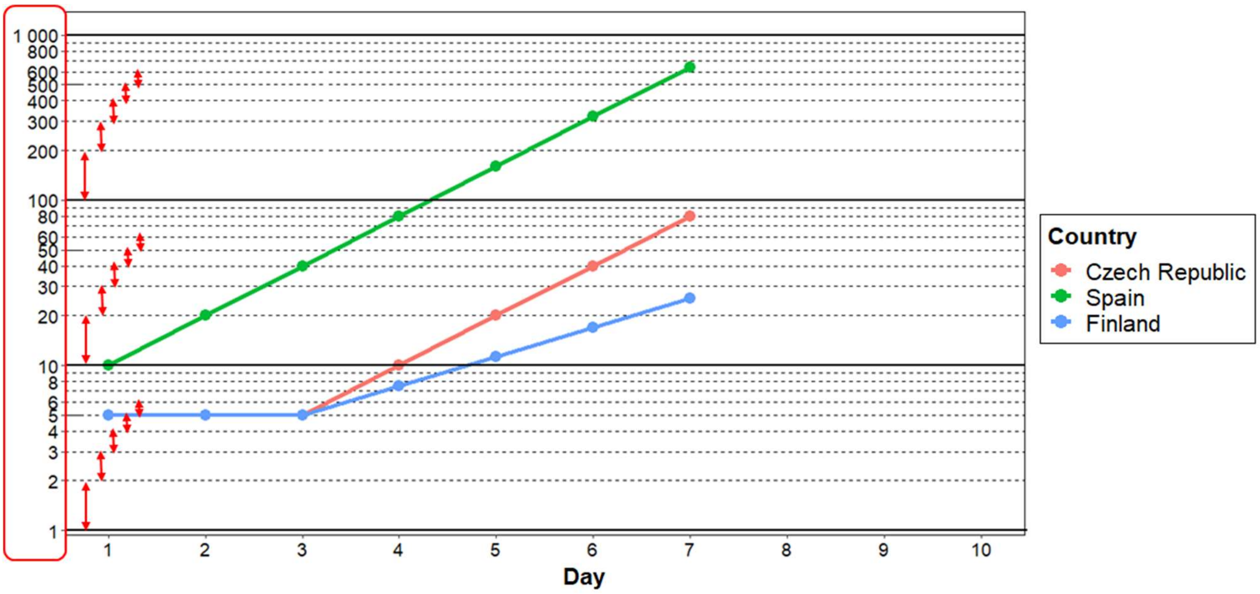

2.2 Linear scale condition (translated from Lithuanian)

Take a closer look at the numbers of cases in the Czech Republic compared to Spain and Finland. Note that the growth can differ not only in its rate (slow or fast), but also in its starting point (starting from the very beginning at a fast rate or starting slower and increasing in rate later).

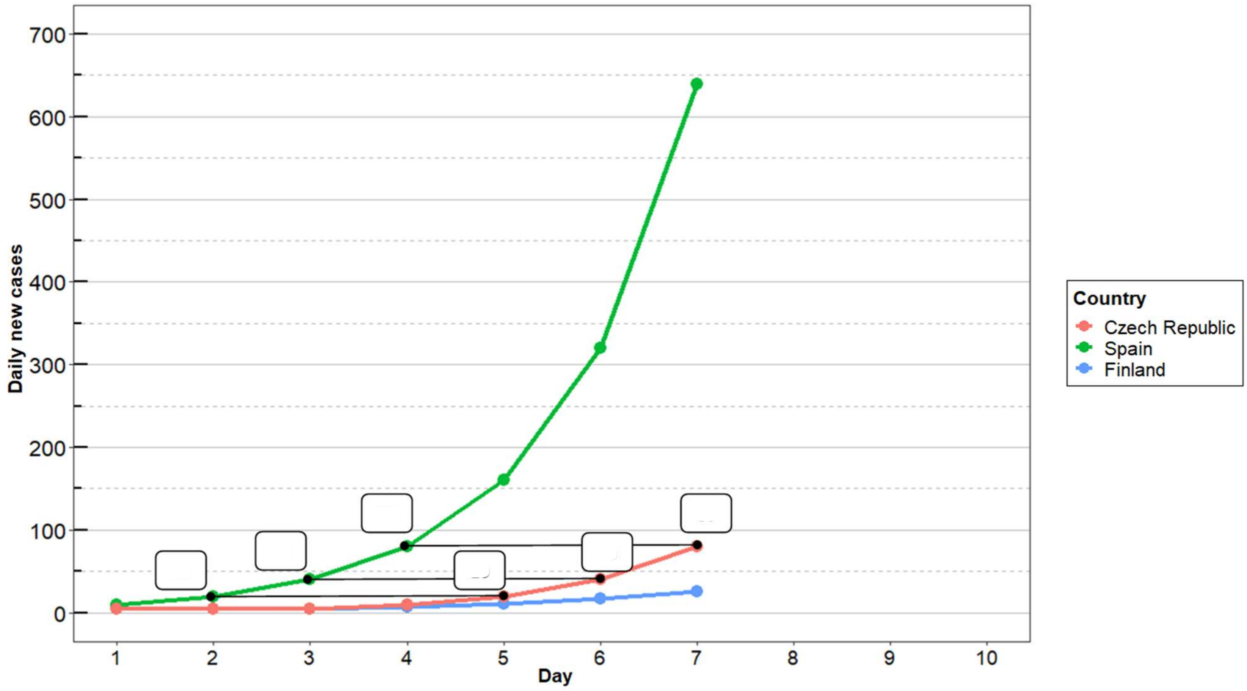

For example, the number of cases in the Czech Republic and Spain are the same with a 3-day gap. For example: on the 3rd day in Spain, and on the 6th day in the Czech Republic, there were 40 new cases. This means that the number of new cases in the Czech Republic reaches the number of cases in Spain after 3 days.

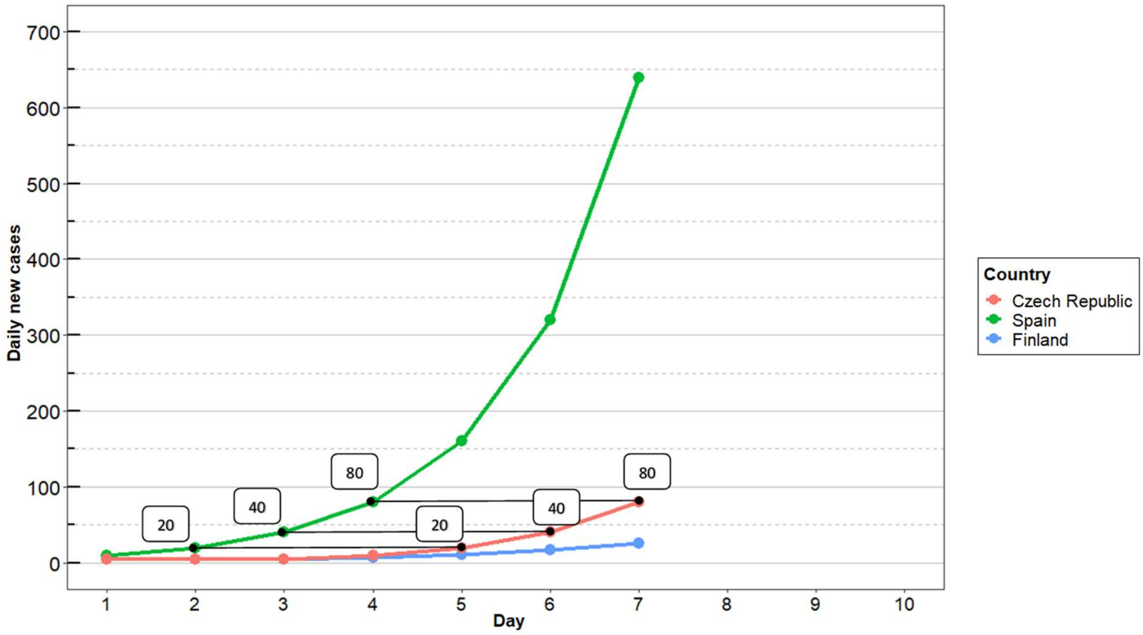

Supplement: Supplementary file 1 [file Data_Sheet_1.pdf]
